# UBE2T promotes nasopharyngeal carcinoma cell proliferation, invasion, and metastasis by activating the AKT/GSK3β/β-catenin pathway

**DOI:** 10.18632/oncotarget.7805

**Published:** 2016-03-01

**Authors:** Wei Hu, Lushan Xiao, Chuanhui Cao, Shengni Hua, Dehua Wu

**Affiliations:** ^1^ Department of Radiation Oncology, Nanfang Hospital, Southern Medical University, Guangzhou, China; ^2^ Department of Infectious Diseases and Hepatology Unit, Nanfang Hospital, Southern Medical University, Guangzhou, China

**Keywords:** nasopharyngeal carcinoma, UBE2T, proliferation, metastasis, AKT/GSK3β/β-catenin pathway

## Abstract

Increasing evidence has shown that UBE2T plays an important role in genomic integrity and carcinogenesis; however, its role in nasopharyngeal carcinoma (NPC) has not been investigated. Here, we evaluated the clinicopathological significance of UBE2T in NPC and its underlying mechanisms. Using immunohistochemical analysis of UBE2T expression in NPC samples, we demonstrated that UBE2T is highly expressed in NPC tissues, which correlated with the T/M classification, skull invasion, and poor prognosis. The *in vitro* assay showed that UBE2T overexpression promoted proliferation, migration, and invasion of NPC cells, while UBE2T knockdown inhibited these processes. Consistent with our *in vitro* results, *in vivo* studies indicated that UBE2T overexpression promoted the growth of NPC xenografts and NPC cell metastasis. We found that UBE2T overexpression activated, whereas UBE2T knockdown inhibited, the AKT/GSK3β/β-catenin pathway. Moreover, the pathway-activation and *in vitro* pro-metastasis effects of UBE2T were blocked by the AKT inhibitor, MK-2206 2HCl. Additionally, UBE2T and p-GSK3 β co-expressed in NPC samples by serial section, and their expressions are correlated. Collectively, our findings demonstrated that UBE2T is a possible diagnostic/prognostic biomarker for NPC and may promote the development and progression of NPC by activating the AKT/GSK3β/β-catenin pathway. Thus, UBE2T could serve as an alternative target for the treatment of NPC.

## INTRODUCTION

Nasopharyngeal carcinoma (NPC) is one of the most common forms of cancer in Southern China, with an incidence of up to 25 per 100,000 individuals per year [1]. NPC is highly malignant, characterized by rapid growth and early distant metastasis [2, 3]. With the development of precise radiation technology, the 5-year overall survival rate of patients with NPC has increased to between 53% and 80% [4]. Regrettably, a few patients still relapse soon after therapy, while others are diagnosed with distant metastasis, do not qualify for curative treatment and experience poor prognosis [5]. However, the specific development and progression mechanisms in NPC have not yet been completely understood [6]. Therefore, to improve the prognosis of NPC there is an urgent need to clarify underlying mechanisms, identify diagnosis/prognosis-related biomarkers, and determine new therapeutic targets for treatment.

Ubiquitin-conjugating enzyme E2T (UBE2T, GenBank accession no. AB032931) functions by combining with specific E3 ubiquitin ligase for degradation or functional changes of corresponding substrate [7]. It was first identified in a case of Fanconi anemia, which is characterized by bone marrow failure and predisposition to development of acute myeloid as well as head and neck squamous carcinomas, and is considered to be mainly caused by the dysregulation of a DNA damage-repair pathway, called the Fanconi anemia (FA) pathway [8–10]. UBE2T is a component of this pathway. Recent studies have shown that disruption of UBE2T expression could directly lead to Fanconi anemia as well as an increase in tumor cell sensitivity to crossing-link agents, by interfering with the DNA damage-repair response [10, 11]. Additionally, overexpression of UBE2T is known to promote mammary carcinogenesis [12]. Considering the important role that UBE2T plays in genomic integrity and carcinogenesis, we investigated if UBE2T is involved in the development and progression of NPC.

In the present study, to analyze the correlation between UBE2T expression and clinical parameters, immunohistochemistry (IHC) of UBE2T in specimens from 149 patients with NPC was performed. Proliferation, invasion, and metastasis assays *in vitro* and *in vivo* were performed to determine the functions of UBE2T. Western blot and immunofluorescence were used to determine possible mechanisms. Our findings suggest that UBE2T is not only a potential biomarker but may also serve as an alternative therapeutic target for NPC.

## RESULTS

### UBE2T expression was correlated with malignant characteristics and outcome of NPC patients

**Figure 1:**
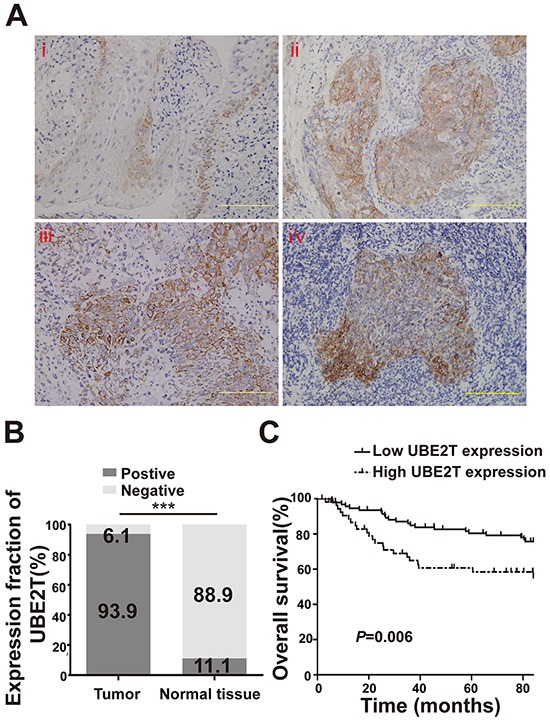
UBE2T expression in NPC patient samples by IHC **A.** Representative image of normal nasopharyngeal mucosal tissue (i) Representative images of nasopharyngeal carcinoma tissue (ii and iii) and a typical cancer nest (iv). Scales indicate 100 μm. **B.** UBE2T expression in tumor tissue and adjacent normal tissue by IHC (Chi-square test, ***P<0.001). **C.** Kaplan–Meier survival curve of 149 patients based on the level of UBE2T expression in tumor tissue.

To investigate UBE2T expression in NPC tissues, we evaluated UBE2T levels in paraffin-embedded samples from 149 patients with NPC by IHC. UBE2T was variably expressed in the cytoplasm of tumor cells in 140 out of the 149 samples, with higher expression in the peripheral region than in the central region of the typical cancer nest. However, only weak expression was noted in 10 out of the 90 samples of adjacent normal tissue, especially in the basilar membrane cells of normal nasopharyngeal mucosa. Representative images are shown in Figure 1A. Chi-square analysis showed that the UBE2T positive-expression ratio in tumor tissue was higher than that in adjacent normal tissue (Figure 1B; *P*<0.001). Next, we analyzed the association between UBE2T expression in tumor tissue and clinical pathological parameters of patients with NPC. UBE2T expression was positively correlated with the T classification (*P*=0.006), M classification (*P*=0.038), and skull base invasion (*P*=0.022); however no correlation was observed with gender, age, clinical classification, and recurrence (Table 1). Meanwhile, we found that patients whose tumor tissue showed high UBE2T expression had shorter overall survival time than those with low UBE2T expression, using Kaplan–Meier analysis and log-rank test (Figure 1C; *P*=0.006). More importantly, univariate and multivariate Cox regression analysis indicated that N/M classification, skull base invasion, and UBE2T expression were each, recognized as independent prognostic factors in patients with NPC (Table 2).

**Table 1:** Correlation between UBE2T protein expression and clinical parameters in NPC

Clinical parameter	N	UBE2T expression		*P*-value
		Low	high	
Gender				
Male	109	68	41	1.000
Female	40	25	15	
Age				
≤45	67	45	22	0.311
>45	82	48	34	
Clinical classification				
I-II	77	53	24	0.127
III-IV	72	40	32	
T classification				
T1-T2	101	71	30	0.006*
T3-T4	48	22	26	
N classification				
N0	94	56	38	0.602
N1-3	55	37	18	
M classification				
M0	131	86	45	0.038*
M1	18	7	11	
Skull base invasion				
No	125	83	42	0.022*
Yes	24	10	14	
Recurrence				
No	136	85	51	0.945
Yes	13	8	5	

* significance as indicated

**Table 2:** Summary of survival analyses by multivariate Cox regression analysis

	Univariate analysis				Multivariate analysis			
	*P*	Ex (B)	95% CI		*P*	Exp (B)	95% CI	
Gender	0.051	0.556	0.309	1.003				
Age	0.215	1.420	0.816	2.471				
T classification	0.050	1.741	1.000	3.030				
N classification	0.000*	2.745	1.591	4.738	0.000*	3.356	1.833	6.147
M classification	0.000*	7.273	3.983	13.280	0.000*	5.258	2.622	10.545
Clinical classification	0.000*	3.054	1.710	5.455	0.137	1.676	0.848	3.314
Skull base invasion	0.003*	2.578	1.371	4.847	0.043*	2.217	1.024	4.800
Recurrence	0.002*	2.944	1.470	5.898	0.146	1.738	0.824	3.664
Expression of UBE2T	0.009*	2.074	1.201	3.581	0.030*	1.933	1.065	3.509

* significance as indicated

### UBE2T promoted NPC cell proliferation *in vitro* and *in vivo*

**Figure 2:**
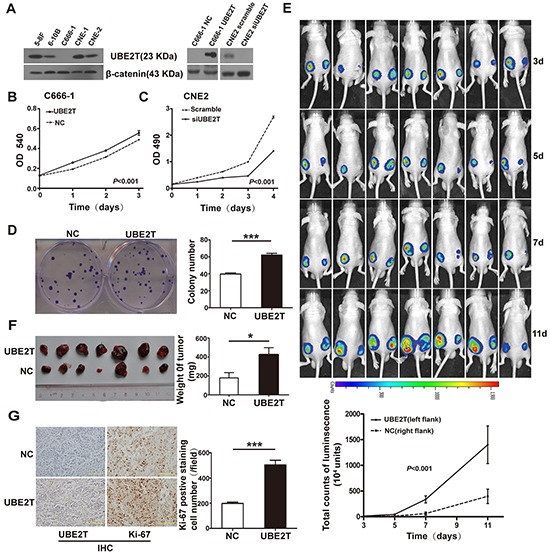
UBE2T promoted NPC cell proliferation *in vitro* and *in vivo* **A.** Background expression of UBE2T in NPC cell lines was determined by western blotting (left). UBE2T overexpression or knockdown in NPC cells was verified at 48 hours after transfection by western blotting (right). **B.** and **C.** MTT assay determined the effects of UBE2T overexpression on C666-1 cell proliferation capacities (B). CCK-8 assay determined the effect of UBE2T knockdown on CNE2 cell proliferation capacities (C). Graph shows the mean ± standard error of mean (SEM) of absorbance at different time points (n=5 or 3, analysis of variance [ANOVA] of factorial design). **D.** Representative images of colony formation assay (left). The bar chart represents mean ± SEM values for number of colonies from indicated groups (n=3, Student's *t*-test, ***P<0.001). **E.** Images of nude mice at indicated time points after subcutaneously injecting C666-1 cells as shown, using the IVIS Lumina II system (up; left flank: UBE2T, right flank: normal control [NC]). The graph shows mean ± SEM values of luminescence signal intensities from both flanks of nude mice at indicated time points (down; n=7, Repeated ANOVA). **F.** Theimages of indicated C666-1 subcutaneous xenografts (left). The graph shows mean ± SEM values of xenograft weight (n=7, Student's *t-*test, *P=0.024). **G.** Representative images of UBE2T and ki-67 expression in C666-1 xenografts from indicated groups by IHC (left). Scales indicate 100 μm. The graph shows mean ± SEM values for the number of ki-67-positive cells from 5 random 40X objective fields for each group (right; Student's *t*-test, ***P<0.001).

To investigate the biological function of UBE2T in NPC, background expression of UBE2T was determined in a panel of NPC cells by western blot. UBE2T was highly expressed in 5-8F, 6-10B, CNE1, and CNE2 cell lines, but showed relatively low expression in C666-1 cells (Figure 2A). Subsequently, UBE2T-overexpressing C666-1 cells were established by transfecting UBE2T lentivirus (UBE2T), while UBE2T-knockdown CNE2 cells were established by disrupting endogenous UBE2T with small interfering RNA (siUBE2T) (Figure 2A). We found that UBE2T-overexpressing C666-1 cells showed a significantly higher proliferation rate than empty vector-transfected control (NC) using the MTT assay (Figure 2B; *P*<0.001). Conversely, UBE2T knockdown inhibited the proliferation of CNE2 cells compared to scrambled UBE2T (Scramble) using the CCK8 assay (Figure 2C; *P*<0.001). In addition, UBE2T promoted colony formation of C666-1 cells compared to the control (Figure 2D; *P*<0.001). These data suggest that UBE2T promoted NPC cell proliferation *in vitro*. To verify this pro-proliferation activation of UBE2T *in vivo*, we subcutaneously inoculated UBE2T- and luciferase-co-expressed C666-1 cells into nude mice. Changes in the luminescence absorption value of xenografts indicated that xenografts overexpressing UBE2T showed faster growth than the control (Figure 2E; *P*<0.001). Moreover, the tumor xenografts were larger and heavier than the control (Figure 2F; *P*=0.024). The number of cells showing Ki-67 positivity (a proliferation-related marker) in xenografts over expressing UBE2T was much higher than in the control (Figure 2G; *P*<0.001). Nevertheless, silencing UBE2T using shRNA lentivirus (shUBE2T) inhibited the growth of CNE2 subcutaneous xenografts compared to the control (Supplementary Figure S1A and S1B, *P*<0.001). Overall, these findings indicate that UBE2T could facilitate NPC cell proliferation *in vitro* and *in vivo*.

### UBE2T enhanced invasive and metastatic capacities of NPC cells *in vitro* and *in vivo*

**Figure 3:**
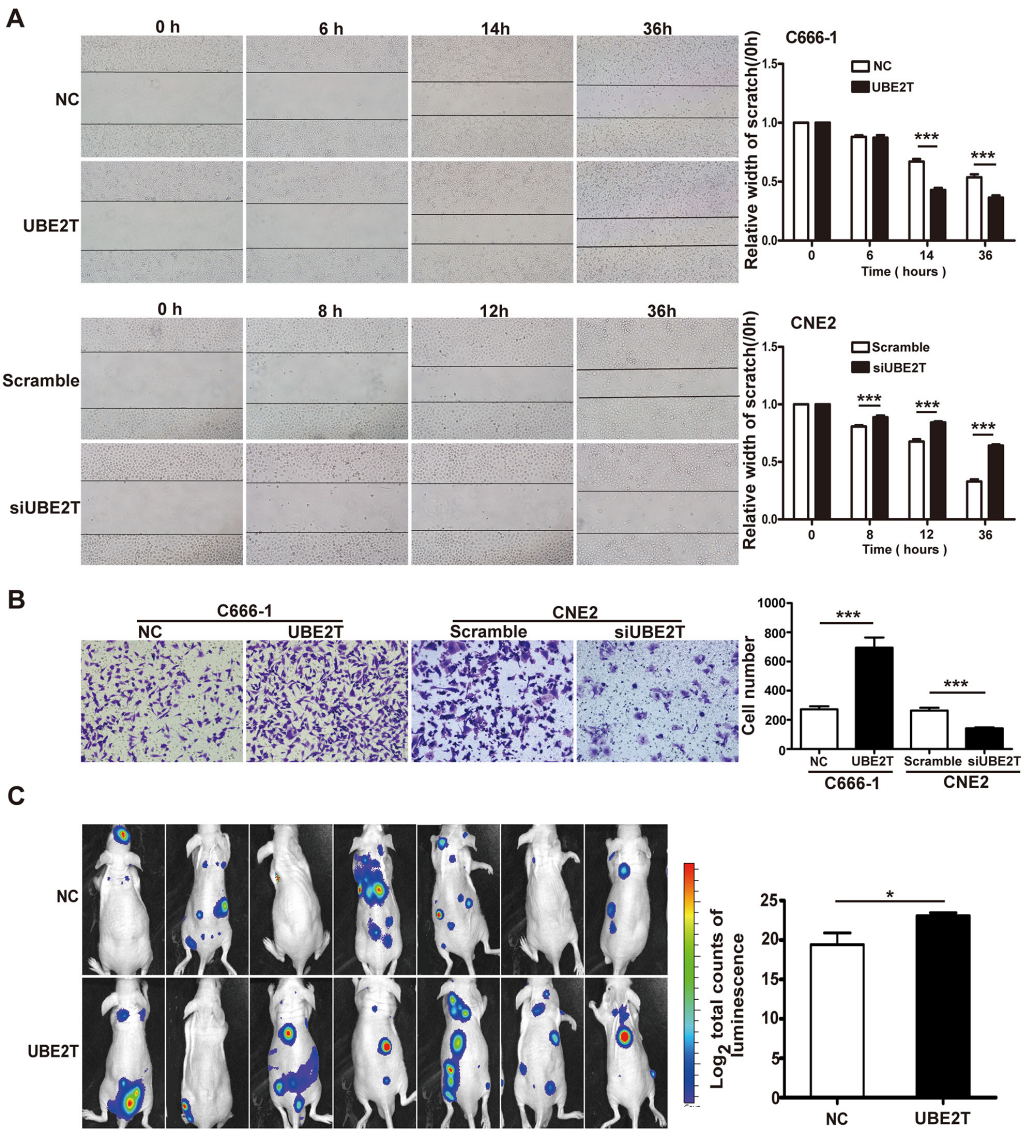
UBE2T promotes NPC cell migration, invasion, and metastasis *in vitro* and *in vivo* **A.** Representative images of scratch assay from indicated groups at different time points (left); The bar chart represents mean ± SEM of relative scratch width (relative to that at 0 hour width; n=5, ANOVA, *P<0.05, and ***P<0.001). **B.** Representative images of the matrix-coated transwell assay from indicated groups (left). The bar chart represents mean ± SEM number of invasive cells from 5 random 20X objective fields (Student's *t*-test, ***P<0.001). **C.** Images of nude mice photographed with IVIS Lumina II system at 17 days after injecting C666-1 cells in the tail vein. The bar chart represents mean ± SEM of log_2_ total luminescence signal intensities (n=7, Student's *t*-test, *P=0.034).

We found that UBE2T high expression correlated with skull base invasion and metastasis in patients with NPC. Next, we investigated whether UBE2T promoted NPC cell invasion and metastasis. Using scratch and matrix-coated transwell assays, we found that over-expression of UBE2T significantly promoted C666-1 cell migration and invasion (Figure 3A and 3B), whereas UBE2T knockdown inhibited CNE2 migration and invasion *in vitro* (Figure 3A and 3B). Further *in vivo* studies showed that UBE2T improved C666-1 cell metastasis in nude mice, as indicated by the total luminescence absorption in tumor cells (total luminescence absorption = the sum of all absorption values) [13] (Figure 3C; *P*=0.034). Verified by hematoxylin-eosin staining, metastasis in the UBE2T overexpressed group was higher than that in the control group (Supplementary Figure S2A and S2B). These results demonstrate that invasive and metastatic capacities of NPC cells were enhanced by UBE2T *in vitro* and *in vivo.*

### UBE2T promotes NPC cell proliferation and metastasis by activating AKT/GSK3β/β-catenin pathway

**Figure 4:**
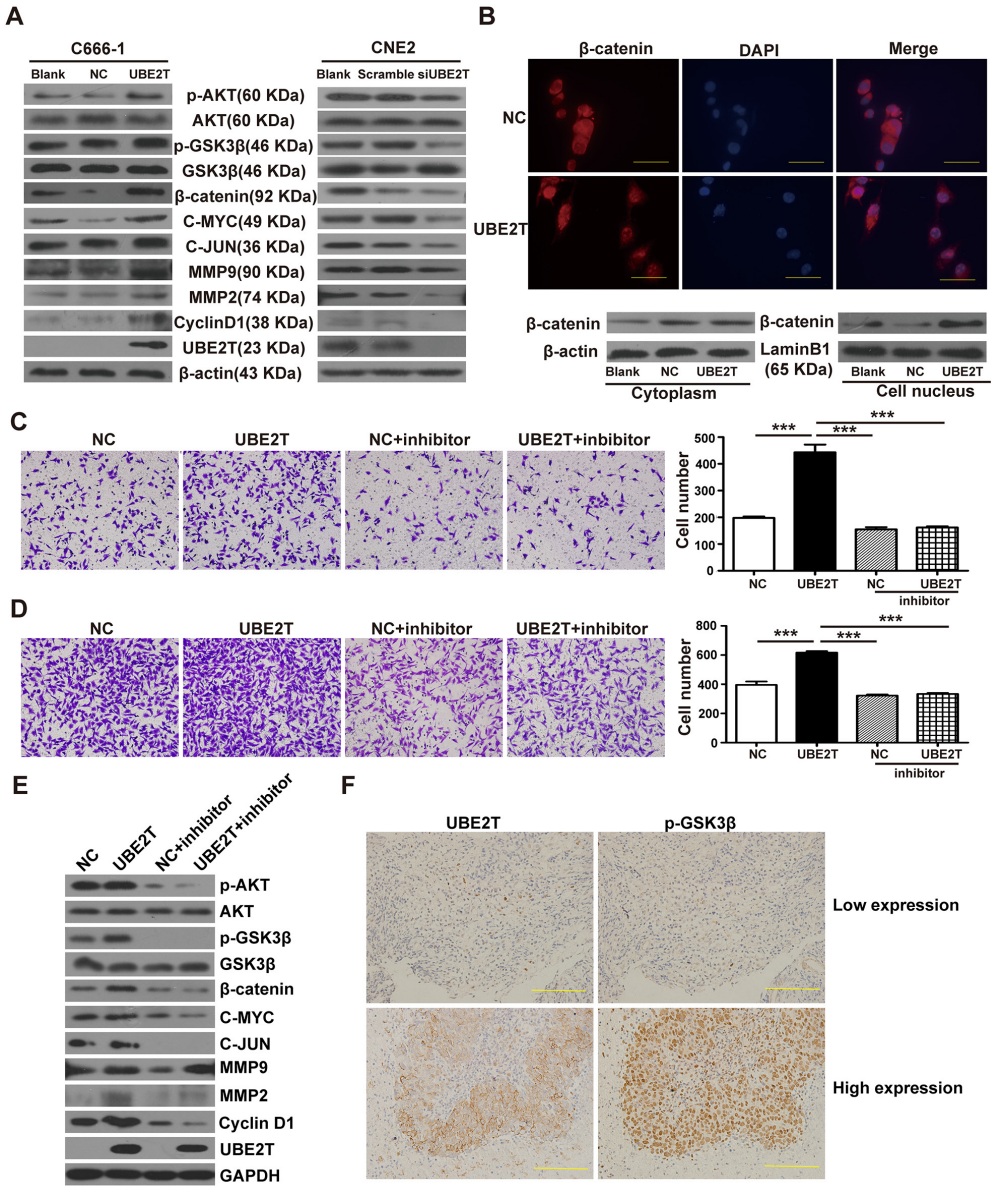
UBE2T promotes NPC cell proliferation and metastasis probably by activating the AKT/GSK3β/β-catenin pathway **A.** Western blot detected the effects of UBE2T on β-catenin, its downstream proliferation/metastasis-related target proteins (Cyclin D1, C-MYC, C-JUN, MMP2, and MMP9), and its upstream pathway proteins (p-AKT, p-GSK3β). **B.** Immunofluorescence determined the effects of UBE2T overexpression on nuclear translocation of β-catenin. Scales indicate 40 μm. (up; ×1000 field), and separate nuclear and cytoplasmic protein western blot verified the effects of UBE2T on nuclear translocation of β-catenin (down). **C.** and **D.** Transwell and matrix-coated transwell analysis detected the effects of AKT inhibitor (MK-2206 2HCl) on the pro-migration and invasion abilities. Representative images of the transwell (C) and matrix-coated transwell (D) assay from indicated groups at 6h (migration) and 24h (invasion). The bar chart represents mean ±SEM number of migration and invasive cells from 5 random 20X objective fields (analysis of variance [ANOVA] of factorial design, ***P<0.001). **E.** Western blotting detected the effects of AKT inhibitor (MK-2206 2HCl) on AKT/GSK3β/β-catenin pathway. **F.** UBE2T and P-GSK3β co-expression analysis in the additional NPC samples through serial section technique. Scales indicate 100 μm.

β-Catenin is an important tumor-associated transcription factor, which plays a pivotal role in tumor cell proliferation and metastasis through its aberrant expression and nuclear translocation [14–16]. We explored whether UBE2T promoted NPC cells proliferation and metastasis by mediating the β-catenin pathway. We found that UBE2T overexpression in C666-1 elevated protein levels of β-catenin and promoted β-catenin downstream proliferation/metastasis-related target proteins CyclinD1, C-MYC, C-JUN, MMP2, and MMP9 expression, while increasing upstream phosphorylated AKT and GSK3β levels, as compared to the control, by western blot analysis (Figure 4A). On the other hand, UBE2T knockdown resulted in decrease of AKT/GSK3β/β-catenin pathway-related protein expression in CNE2 cells. Additionally, immunofluorescence staining results demonstrated that UBE2T promoted the accumulation of β-catenin in the nucleus of C666-1 cells; this was verified by separate western blot analysis of nuclear and cytoplasmic proteins (Figure 4B). MK-2206 2HCl (a specific inhibitor of AKT) blocked the pro-migration/invasion effects (all *P* < 0.001) and the activating of AKT/GSK3β/β-catenin pathway resulted from UBE2T overexpression, as confirmed via transwell analysis (Figure 4C and 4D) and western blot (Figure 4E). Collectively, these results suggest that UBE2T might promote NPC cell proliferation and metastasis via modulating the AKT/GSK3β/β-catenin pathway. To validate this conclusion, we performed IHC analysis on serial sections of 20 additional NPC samples for UBE2T and p-GSK3β expression. The result showed the UBE2T and p-GSK3β were co-expressed in these samples (Figure 4F), and their expressions are correlated (Supplementary Figure S3, *P* = 0.007).

## DISCUSSION

In this study, we report that UBE2T is mainly expressed in NPC tissues and that this expression is correlated with the T/M classification, skull base invasion, and poor prognosis of NPC. More importantly, UBE2T is an independent prognostic factor for NPC and promoted proliferation, invasion, and metastasis of NPC by activating the AKT/GSK3β/β-catenin pathway.

Cumulative evidence suggest that UBE2T is an essential component of the FA pathway, playing a critical role in maintaining integrity of the genome [10, 17]. Recent studies have shown that UBE2T is highly expressed in tumor tissues and promotes carcinogenesis, implicating that UBE2T plays a role in the malignant tumor phenotype [12, 18]. The results of this study clarify this point further. We found that UBE2T was not only involved in the malignant phenotype of patients with NPC but was also an independent prognostic factor for NPC. Hence, UBE2T can be considered as a diagnostic/prognostic biomarker of NPC. Nevertheless, prospective clinical studies are still needed to confirm the clinical value of UBE2T.

Previous studies have revealed that UBE2T promotes colony formation in NIH3T3 cells and that knockdown of endogenous UBE2T inhibited proliferation of T47D and BT-20 breast cancer cell lines [12, 18]. This is consistent with our results that UBE2T promotes colony formation and proliferation of NPC cells *in vitro* and *in vivo*. Additionally, we found that over expressing UBE2T promoted NPC cell migration, invasion, and metastasis *in vitro* and *in vivo*, whereas, knockdown of UBE2T inhibited these effects *in vitro*. We believe that this is the first report of pro- invasion and metastasis effects of UBE2T in NPC. These results suggested that UBE2T is a crucial cancer-promoting gene involved in the development and progression of NPC. This is in accordance with our clinical observation that high UBE2T expression level is positively correlated with aggressive characteristics and poor prognosis of patients with NPC.

Mechanistically, CyclinD1, C-JUN, C-MYC, MMP2, and MMP9 are well-known pro-proliferation/metastasis proteins [19–23]. This could explain the pro-proliferation and metastasis effects of UBE2T. Brand et al. showed that UBE2T directly interacts with BRCA1/BARD1, resulting in its proteasomal degradation, which promotes proliferation of breast cancer cells [12]. Wen et al. found that UBE2T exhibits oncogenic properties in human prostate cancer and suggested that UBE2T overexpression was sufficient to induce epithelial-mesenchymal transition of PCa cells as well as promote tumor growth and metastasis by cooperating with vimentin [24]. Given that UBE2T is a E2 ubiquitin-conjugating enzyme lacking specificity for substrates and has versatile roles [12, 18, 24, 25], we hypothesized that UBE2T would possess a context-dependent role in different cancer types, wherein UBE2T functions through different substrates and signal pathways to facilitate the development and progression of NPC. AKT/GSK3 β/β-catenin pathway is known to be involved in cell proliferation, survival, EMT, and metastasis in some malignant tumors including NPC [26, 27]. β-catenin functions as a key transcriptional factor regulating the expression of pro-proliferation/metastasis proteins (CyclinD1, C-JUN, C-MYC, MMP2, and MMP9) via aberrant expression and nuclear translocation [27–31]. Coincidently, in our study we found that over expressing UBE2T increased β-catenin expression and elevated levels of p- AKT and p-GSK3β, while promoting β-catenin nuclear translocation. On the other hand, UBE2T knockdown resulted in the opposite effect. Moreover, MK-2206 2HCl (a specific inhibitor of AKT) blocked the pro-migration/invasion and pathway-activating effects of UBE2T overexpression in NPC cells. These results suggest that UBE2T promotes NPC proliferation and metastasis, probably by activating AKT/GS3KB/β-catenin pathway. In addition, UBE2T and p-GSK3β existed co-expression and correlation in NPC samples, which further validated our speculation. Considering that UBE2T regulates both, proliferation and invasion/metastasis through such a significant pathway and ubiquitination inhibitors have shown positive results in clinical trials [32, 33], we emphasize that UBE2T could be a potential therapeutic target for NPC. Further studies to identify the protein that directly binds to UBE2T and activates the AKT/GSK3β/β-catenin pathway in NPC will not only further elucidate the mechanism of UBE2T activity, but also provide a novel therapeutic target for NPC. This could lead to development of a new treatment strategy involving disruption of the interaction between UBE2T and AKT/GSK3β/β-catenin pathway.

In conclusion, we suggest that UBE2T can be considered as a diagnostic and prognostic biomarker as well as an novel therapeutic target for NPC.

## MATERIALS AND METHODS

### Patients

The study included paraffin-embedded samples from 149 consecutive patients diagnosed with NPC and treated primarily between 1996 and 2000 at Nanfang Hospital, Southern Medical University. An additional paraffin-embedded samples of 20 patients diagnosed with NPC in 2015 at Affiliated Hospital of Zunyi Medical College were used in this study for co-expression analysis; these samples were devoid of clinical and follow-up information. Informed consent was obtained from patients to permit use of their tissue samples and data for research, as required by the Southern Medical University Ethics Committee. For the 149 patients with NPC, the cancer stage was determined as per the 1992 Fuzhou NPC staging system of China (Supplementary Table S1) [34]. Of these, 109 (73.1%) patients had previously received radiotherapy alone and 40 (26.8%) had received combined chemoradiotherapy. Response to radiotherapy was evaluated at 3 months after completion of treatment, by clinical examination, computed tomography scan, and biopsy. The clinicopathological characteristics and UBE2T expression in all 149 patients are described in Supplementary Table S2. All these patients were followed up for 7 years.

### Immunohistochemical staining (IHC)

Formalin-fixed, paraffin-embedded, 4-μm tumor sections were rehydrated in a graded series of alcohol. Endogenous peroxidase activity was blocked with 3% H_2_O_2_. Subsequently, slides were steamed for 3 minutes for antigen retrieval in 10 mM citrate buffer (pH 6.0) and incubated with primary antibody at 4°C overnight, and with secondary antibody (Dako) for 1 hour. The sections were stained with 3,3′-diaminobenzidine tetrahydrochloride (DAB; Dako, Glostrup, Denmark) to visualize the stained areas under a light microscope (Olympus, Tokyo, Japan) and counterstained with hematoxylin. The immunoreactive score (IRS) of UBE2T was determined as previously described [34]. Briefly, the intensity of staining was scored as 0 (negative), 1 (weak), 2 (medium), or 3 (strong). The extent of staining was scored as 0 (0%), 1 (1% – 25%), 2 (26% – 50%), 3 (51% – 75%), or 4 (76% – 100%), according to the percentage of positively stained areas in the tumor tissue. The sum of the intensity and extent scores was used as the total IRS (0–7) of UBE2T. A total IRS of <=3 was considered as low UBE2T expression, whereas that of >3 was considered as high UBE2T expression. UBE2T scoring was evaluated individually and independently by 2 senior pathologists, blinded to the clinical data. The number of ki-67-positive staining cells was counted in 5 random 40X objective fields of IHC samples from subcutaneous xenografts to evaluate the pro-proliferation capacity of UBE2T *in vivo*. Primary antibodies used were UBE2T (#10105-2-AP, 1:200, proteintech, Chicago, IL, USA), Ki-67 (# ZM-01666,1:100, ZSGB-BIO, Beijing, China), and p-GSK3 β (#9322, 1:100, CST, Boston, MA, USA). The co-expression and correlation analysis of UBE2T and p-GSK3 β was performed using serial section technique.

### Cell culture

C666-1, 5-8F, 6-10B, CNE1, and CNE2 NPC cell lines from the Cancer Research Institute of South Medical University were cultured in Dulbecco's modified Eagle's medium (Gibco, Grand Island, NY, USA) supplemented with 10% fetal bovine serum (Gibco). All cell lines were cultured at 37°C in a humidified atmosphere with 5% CO_2_.

### Lentiviral transfection

Lentiviral vectors (GENECHEM, Shanghai, China), empty as well as those carrying overexpressed or silenced UBE2T, were transfected into C666-1 or CNE2 cells in the presence of polybrene (GENECHEM, Shanghai, China; 6 mg/ml). After 12 hours, the original medium was replaced with fresh medium. Luciferase lentiviral vectors purchased from GENECHEM (Shanghai, China) were co-transfected with overexpressed UBE2T-carrying or empty vectors for *in vivo* studies as previously described [35]. The efficiency of transfection was verified by western blot and luciferase assay (Promega, Madison, WI, USA) at 48 hours after transfection.

### siRNA transfection

UBE2T was disrupted by small interfering RNA, siUBE2T. siUBE2T oligonucleotides and corresponding scrambled oligonucleotides were purchased from Genepharma (Shanghai, China). Their sequences were as follows: siUBE2T: GCUGACAUAUCCUCAGAAUTT; Scrambled: UUCUCCGAACGUGUCACGUTT. Briefly, CNE2 cells were cultured under complete medium conditions in a 6-well plate, transiently transfected with siUBE2T oligonucleotides and scrambled with 5 μl iMAX (Invitrogen, Carlsbad, CA, USA). After 48 hours, the cells were harvested for western blotting to determine the interfering efficiency.

### *In vitro* proliferation assay

Cell proliferation rates were determined by Cell Counting MTT or CCK-8 assays (Beyotime, Jiangsu, China) according to the manufacturer instructions. Briefly, for the MTT assay, 8×10^2^ UBE2T over-expressing or control C666-1 cells were seeded in 24-well plates. At indicated time points, MTT solution was added to each well and after 4 hours incubation, DMSO was added. The solution was then harvested into 96-well plates and absorbance was read at 540 nm. For CCK8 assay, 1×10^3^ UBE2T-knockdown and -scrambled CNE2 cells were seeded in 96-well plates. At different time points defined as above, CCK-8 reagent in basal medium was added to each well and the absorbance was measured 4 hours later, at 490 nm.

### Colony formation assay

To determine the effects of UBE2T overexpression on colony formation of NPC cells, equal amounts of overexpressed UBE2T-carrying and control C666-1 cells (100 cells/well) were seeded in 6-well tissue culture plates. At 8 days after incubation in a stable incubator, cell colonies were fixed with methanol and stained with 0.1% crystal violet. Colonies with at least 50 cells per well were counted using a light microscope.

### Scratch assay

The effects of UBE2T on migration capacities were assessed using scratch wound assay. A total of 5×10^5^ cells were seeded into 6-well culture plates and cultured to complete confluence. Subsequently, 3 parallel, linear wounds were produced in each dish with a 20-μl plastic pipette tip. The cells were then cultured with serum-free medium. At different time points, 5 representative images of scratched areas from each dish were photographed to estimate migration using Adobe Photoshop 2.0 software (Adobe Systems™, San Jose, California, USA).

### Transwell assay

For measuring the effects of UBE2T on invasion capacities, matrix-coated transwell invasion assay was performed as previously described [36]. Briefly, approximately 1×10^5^ NPC cells were suspended in serum-free medium and added to the top chamber of the 24-well, matrix-coated, transwell chamber system (Corning, Cambridge, MA, USA). Normal cell culture medium was added to the bottom chamber and cells were incubated for 24 hours at 37°C. The membrane was fixed and stained. Cells on the membrane were removed and the number of cells below the membrane were counted (5 random × 200 fields per well). For migration assay, the general transwell chamber was used instead of matrix-coated transwell chamber. Additionally, 2 μM AKT inhibitor (MK-2206 2HCl, S1078, Selleckchem, Houston, TX, USA) was used in the transwell analysis.

### Western blot

Total proteins were extracted using RIPA lysis buffer (Beyotime, Jiangsu, China) added along with protease inhibitor and phosphoesterase inhibitor (Roche, Basel, Switzerland). Cellular fractionation was performed as previously described [37]. Total protein was quantified using the Bradford method (Bio-Rad Laboratories, Hercules, CA, USA). Total protein samples (20 μg) were subjected to sodium dodecyl sulfate-polyacrylamide gel electrophoresis and the proteins were transferred to polyvinylidene fluoride membranes (Bio-Rad Laboratories). Next, the membranes were incubated with 5% bovine serum albumin at room temperature for 1 hour. The membranes were then incubated with primary antibodies at 4°C overnight. After 3 washes with Tris-buffered saline with Tween (TBST), the membranes were incubated with horse-radish peroxidase (HRP)-conjugated rabbit or mouse secondary antibodies for 1 hour at room temperature (Abcam, Cambridge, MA, USA). Triple-washed with TBST, bands were visualized using enhanced chemiluminescence (ECL) reagents (Thermo Fisher Scientific, Rockford, IL, USA). Primary antibodies used are as follows: anti-β-catenin (ab32572, 1:7000) and anti-MMP9 (ab76003, 1:5000) purchased from Abcam; anti-UBE2T (#10105-2-AP, 1:1000) from Proteintech; Cyclin D1 (sc753, 1:500) was obtained from Santa Cruz Biotechnology (Santa Cruz, California, USA); β-actin (AP0060, 1:4000), C-MYC (BS2462, 1:500) and MMP2 (BS1236, 1:1000) were obtained from Bioworld Technology (Louis Park, MN, USA); C-JUN (A0246, 1:1000) was purchased from ABclonal (Cambridge, MA, USA); AKT (#4691,1:1000), p-AKT (Ser473) (#4060, 1:2000), GSK3β (#9336, 1:1000), and p-GSK3β (#9322, 1: 1000) were obtained from CST (Boston, MA, USA). β-actin and luminB1 were used as loading control. Additionally, 2 μM MK-2206 2HCl was used in the western blot analysis.

### Immunofluorescence

NPC cells were stained by immunofluorescence on coverslips. Briefly, cells were washed with phosphate-buffered saline (PBS), fixed in 4% paraformaldehyde for 10 minutes, and permeabilized with 0.25% Triton X-100 for 5 minutes, followed by 3-hour incubation with β-catenin antibodies (#ab32572, 1:200), triple washing with PBS, and incubation with cy3-conjugated CA goat antibodies against rabbit IgG (ZSGB-BIO, Beijing, China) for 1h. The coverslips were counterstained with 4,6-diamidino-2-phenylindole (ZSGB-BIO, Beijing, china). The observations were noted and photographed using a fluorescence microscope with 100X objective (Olympus, Tokyo, Japan).

### Proliferation and metastasis analysis *in vivo*

NPC cells were co-transfected with firefly luciferase lentivirus and UBE2T lentivirus or corresponding empty vector. For *in vivo* proliferation analysis, 5×10^5^ cells were injected subcutaneously into bilateral flanks of nude mice to establish tumors. For metastasis analysis, 1×10^6^ cells were injected into the tail vein of nude mice. Next, 4 mg luciferin (Bioworld Technology, Minneapolis, MA, USA) in 50 μl saline was injected intraperitoneally, and 10 minutes later, mice were anaesthetized by inhalation at designated time points and fluorescence absorption was monitored using the IVIS Lumina II system (Caliper, Hopkinton, MA, USA) [35].

In the *in vivo* metastasis assay, suspected lesions of nude mice were sacrificed and dissected under IVIS Lumina II system to detect separated fluorescence absorption. The organs found positive were fixed for confirmation via HE staining. A metastasis organ was defined as one that showed the presence of tumor cells after HE staining by serial section every 1μm. Different lesions from the same organ such as skin, were regarded as a single metastasis organ. Mice were sacrificed 11 days after inoculation for proliferation analysis, whereas mice were photographed and sacrificed at 17 days after inoculation for metastasis analysis. To validated the proliferation inhibitory effects of UBE2T knockdown *in vivo*, we specifically silenced UBE2T by shRNA-lentivirus in CNE2 cells. Subsequently, 2×10^6^ UBE2T Knockdown CNE2 cells(shUBE2T) and the control(NC) were injected into the bilateral flanks of BALB/c nude mice to establish subcutaneous xenograft model. All animal studies were performed according to the institution guidelines for the use of laboratory animals and approved by the Institutional Animal Care and Use Committee of Nanfang Hospital. BALB/c nude mice (4–6 weeks old) were obtained from the Central Laboratory of Animal Science of Southern Medical University.

### Statistical analysis

SPSS 13.0 software (IBM, Armonk, NY, USA) was used for statistical analysis. Data are expressed as mean ± SEM. *P*-value < 0.05 was considered as a statistically significant. The chi-square test was used to analyze the relationship between UBE2T expression and clinicopathological characteristics. Overall survival time was calculated by the Kaplan-Meier method and analyzed by log-rank test. Cox proportional hazard analysis was used for univariate and multivariate analysis to determine the effects of variables on survival. For multivariate analysis, clinical variables were incorporated into the model with inclusion criteria of a *P*-value <0.05 in the univariate analysis. Other statistical methods are described in figure legends.

## SUPPLEMENTARY FIGURES AND TABLES


