# Supplementary material for: UBE2T promotes nasopharyngeal carcinoma cell proliferation, invasion, and metastasis by activating the AKT/GSK3β/β-catenin pathway
Source: Oncotarget. 2016 Mar 1;7(12):15161–72. doi: 10.18632/oncotarget.7805 (PMC4924777; doi:10.18632/oncotarget.7805)
Supplement: Supplementary file 1 [file oncotarget-07-15161-s001.pdf]

SUPPLEMENTARY FIGURES AND TABLES

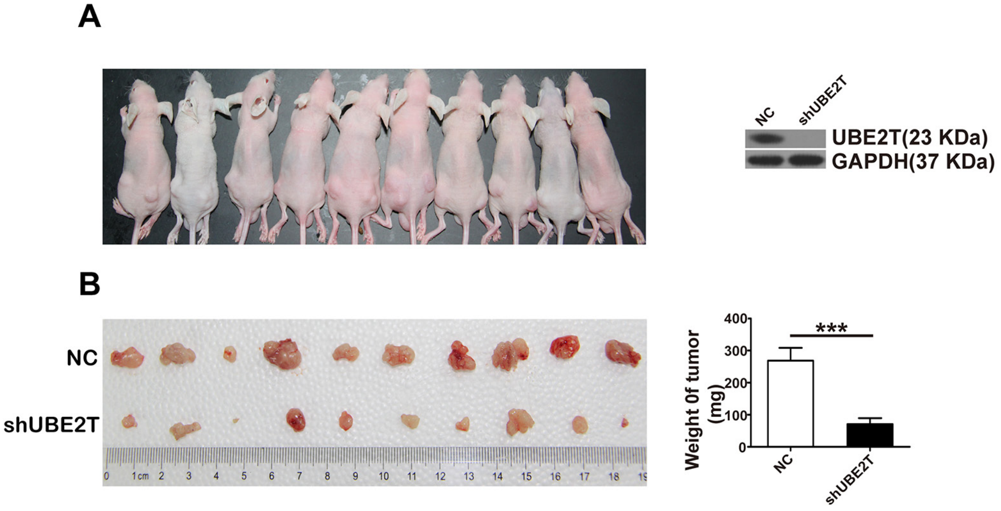

Supplementary Figure S1: Silencing UBE2T with shRNA inhibited the growth of CNE2 subcutaneous xenografts. **A.** Images of nude mice at 10 days after subcutaneously injecting CNE2 cells as shown (left flank: normal control [NC], right flank: shUBE2T). Western blot images show the silence efficiency. **B.** Theimages of indicated CNE2 subcutaneous xenografts (left). The graph shows mean ± SEM values of xenograft weight (n=10, Student's t-test, ***P<0.001).

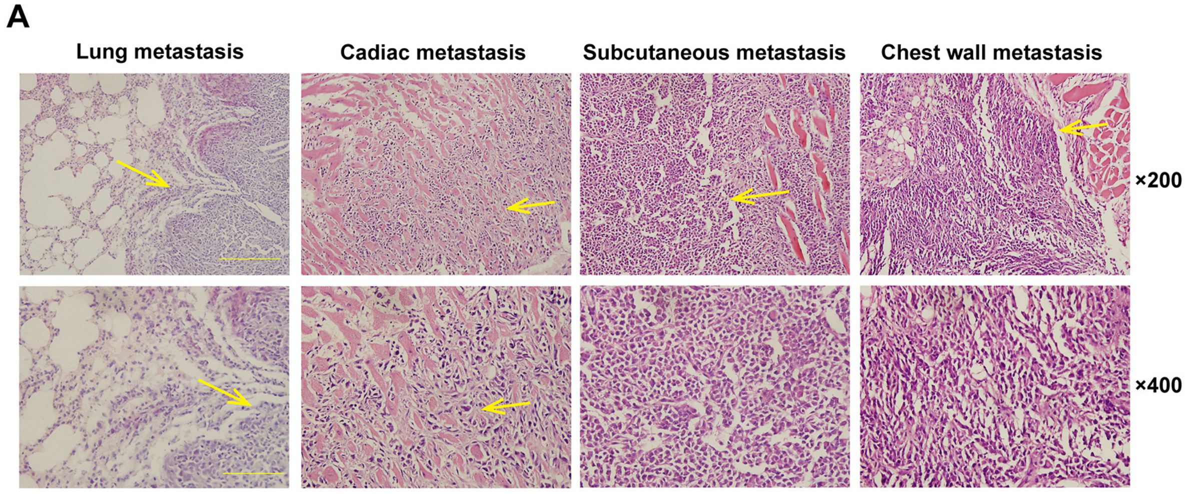

B

Groups	NC	UBE2T
Mice	7	7
Metastatic organs	7	21

Supplementary Figure S2: **A.** Representative images of metastasis under 20/40X objective fields. Scale indicates 200 µm in 20X objective and 100 µm in 40X objective. Yellow arrows indicate lesions. **B.** The number of total metastatic organs verified by HE staining in each group.

	Low UBE2T expression	High UBE2T expression
Low p-GSK3β expression	5	2
High p-GSK3β expression	1	12

Supplementary Figure S3: The correlation analysis of UBE2T and p-GSK3β in 20 additional NPC samples. (chi-square test, P=0.007).

Supplementary Table S1: 1992 Fuzhou staging system for nasopharyngeal carcinoma

**T1:**	Tumor confined to the nasopharynx
**T2:**	Involvement of nasal cavity, oropharynx, soft palatine, anterior cervical vertebrae soft tissue, and parapharyngeal space extension before SO line*
**T3:**	Extension over SO line, involvement of anterior or posterior cranial nerves alone, skull base, pterygopalatine zone, and pterygopalatine fossa
**T4:**	Involvement of both anterior and posterior cranial nerves, paranasal sinus, cavernous sinus, orbit, infratemporal fossa, and direct invasion of first or second cervical vertebra
**N0:**	No enlarged lymph node
**N1:**	Diameter of upper neck lymph node <4 cm, movable
**N2:**	Lower neck lymph node or the diameter between 4 and 7 cm
**N3:**	Supraclavicular lymph node or diameter >7 cm or fixed of skin infiltration
**M0:**	Absence of distant metastasis
**M1:**	Presence of distant metastasis
**Stage I**	T1N0M0
**Stage II**	T2N0-1M0, T1-2N1M0
**Stage III**	T3N0-2M0, T1-3N2M0
**Stage IVa**	T4N0-3M0, T1-4N3M0
**Stage IVb**	Any T, any N, M1

* Line connected from the styloid process to the midpoint on posterior edge of the great occipital foramen; border between upper and lower neck is the lower margin of the cricoid cartilage.

Supplementary Table S2: Clinicopathological parameters of patient samples and UBE2T expression levels in NPC

	N(%)
Gender	
Male	109 (73.2)
Female	40 (26.8)
Age	
≤45	67(45.0)
>45	82(55.0)
T classification	
T1-T2	101 (67.8)
T3-T4	48 (32.2)
N classification	
N0	94 (63.1)
N1-N3	55 (36.9)
M classification	
M0	131 (88.9)
M1	18 (12.1)
Skull base invasion	
No	125 (83.9)
Yes	24 (16.1)
Recurrence	
No	136 (91.3)
Yes	13 (8.7)
Expression of UBE2T	
High expression	56 (37.6)
Low expression	93 (62.4)
